# Screening for hypoglycemia at the bedside in the neonatal intensive care unit (NICU) with the Abbott PCx glucose meter

**DOI:** 10.1186/1471-2431-6-28

**Published:** 2006-11-03

**Authors:** Cynthia Balion, Vijaylaxmi Grey, Afisi Ismaila, Susan Blatz, Wendy Seidlitz

**Affiliations:** 1 Department of Laboratory Medicine, Hamilton Health Sciences, Hamilton, Ontario, Canada; 2 Department of Pathology & Molecular Medicine, McMaster University, Hamilton, Ontario, Canada; 3 Department of Pediatrics, McMaster University, Hamilton, Ontario, Canada; 4 Department of Clinical Epidemiology & Biostatistics, McMaster University, Hamilton, Ontario, Canada; 5 McMaster University Evidence-based Practice Center (EPC), McMaster University, Hamilton, Ontario, Canada; 6 Department of Pediatrics, Hamilton Health Sciences, Hamilton Ontario, Canada

## Abstract

**Background:**

Point of care (POC) glucose meters are routinely used as a screening tool for hypoglycemia in a neonatal setting. Glucose meters however, lack the same accuracy as laboratory instruments for glucose measurement. In this study we investigated potential reasons for this inaccuracy and established a cut off value for confirmatory testing.

**Methods:**

In this prospective study, all patients in the neonatal intensive care unit who had a plasma glucose test ordered were eligible to participate. Demographic information, sample collection information (nine variables) and a recent hematocrit value were recorded for each sample. Glucose measurements were taken at the bedside on the glucose meter (RN PCx) as well as in the laboratory on both the glucose meter (LAB PCx) and the laboratory analyzer (PG). Data were analyzed by simple and mixed-effects regression analysis and by analysis of a receiver operator characteristics (ROC) curve.

**Results:**

There were 475 samples analyzed from 132 patients. RN PCx values were higher than PG values (mean = 4.9%), while LAB PCx results were lower (mean = -5.2%) than PG values. Only 31% of the difference between RN PCx – PG and 46% of the difference for LAB PCx – PG could be accounted for by the variables tested. The largest proportion of variance between PCx and PG measurements was explained by hematocrit (about 30%) with a greater effect seen at glucose concentrations ≤4.0 mmol/L (≤72 mg/dL)(48% and 40% for RN PCx and LAB PCx, respectively). The ROC analysis showed that for detection of all cases of hypoglycemia (PG < 2.6 mmol/L)(PG < 47 mg/dL) the PCx screening cut off value would need to be set at 3.8 mmol/L (68 mg/dL) requiring 20% of all samples to have confirmatory analysis by the laboratory method.

**Conclusion:**

The large difference between glucose results obtained by PCx glucose meter compared to the laboratory analyzer can be explained in part by hematocrit and low glucose concentration. These results emphasize that the glucose meter is useful only as a screening device for neonatal hypoglycemia and that a screening cut off value must be established.

## Background

Adaptation to the extra-uterine environment requires the newborn to establish glucose regulation and this transition usually takes place smoothly. However, some infants such as those that are small or large for gestational age, discordant twins, those with insulin dependent diabetic mothers, or low or extremely low birth weight infants may be at risk for neonatal hypoglycemia [1]. In these patients glucose measurements are usually monitored at the bedside using point of care (POC) glucose meters. In addition to requiring only a small amount of blood compared to laboratory glucose testing, they provide immediate quantitative results allowing for more rapid intervention in cases where low glucose values are found. However, measurements using glucose meters are not as accurate as those taken using laboratory methods and confirmation of low levels are necessary for the diagnosis of neonatal hypoglycemia [2]. While the definition of clinically significant neonatal hypoglycemia is itself controversial and may vary depending on the clinical circumstance [3,4] maintaining therapeutic plasma glucose levels at 2.6 mmol/L or higher is considered desirable [3].

Glucose meters were originally designed for glucose self-monitoring of patients with diabetes and concern has been raised about their accuracy in the neonatal setting [5]. In the neonatal intensive care unit (NICU) agreement between the laboratory value and the bedside reading of ± 0.1 mmol/L or ± 5% at the hypoglycemic level is considered acceptable and should preclude the need for confirmatory or repeat testing [2]. Current POC instruments are unable to achieve this agreement. The current recommendations for analytical quality of glucose meters are those targeted for diabetic patients. The American Diabetes Association (ADA) recommends that glucose meters should have a total error (bias and imprecision) of no more than 10% for all concentrations [6]. More recently, the International Organization for Standardization (ISO) suggested that 95% of measurements should be within ± 0.83 mmol/L for glucose concentrations ≤ 4.2 mmol/L and within ± 20% for concentrations > 4.2 mmol/L [7]. Few studies reporting the performance of glucose meters in the NICU have been able to achieve any of the above criteria or other reasonable accuracy criteria [8-10]. Both pre-analytical (e.g., sample collection, physiologic factors), and analytical factors (e.g., precision) are known to influence the performance of glucose meters but the degree of contribution of individual variables to overall measurement error is poorly understood. For this reason, we undertook a study to assess both the sample characteristics and the pre-analytical parameters that could influence the performance of glucose meter in a neonatal population. At the same time we sought to determine an optimal cut off value for our glucose meter to trigger confirmatory testing.

## Methods

### Subjects

Consecutive patients in the Neonatal Intensive Care Unit (NICU) at the McMaster site of Hamilton Health Sciences who had a plasma glucose test ordered were eligible for inclusion in the study. A glucose meter reliability assessment form was completed for each sample collected. It included demographic information (patient identification number, weight, and corrected gestational age), sample collection information (type of sample, glucose meter identification number, registered nurse identification number, if the first drop of sample was wiped away, if a pipette was used to apply the sample to the glucose meter strip, and if the sample was sent on ice), time data at different stages namely collection, transport, reception and laboratory analysis, and results for the three glucose measurements as well as the most recent hematocrit measurement. The Hamilton Health Sciences/McMaster Faculty of Health Sciences Research Ethics Board approved the study. Parental consent was obtained for the additional testing.

### Glucose measurements

A minimum amount of capillary, arterial, or venous blood was collected in a lithium heparin microtainer. A portion was used for testing glucose at the bedside by a registered nurse using the Precision PCx glucose meter (RN PCx) (Abbott Laboratories, Medisense Products, Bedford, MA) and the remaining sample was sent by a pneumatic tube system to the laboratory for testing by a registered technologist on both the Precision PCx glucose meter (LAB PCx) and the laboratory analyzer (PG) (Vitros 950, Ortho-Clinical Diagnostics, Rochester, NY). Eight different glucose meters were used in the NICU and one glucose meter was used in the laboratory. The same lot number of test strips was used throughout the study. All glucose meters and test strips used in this study were those routinely used in the neonatal wards.

The Precision PCx glucose meter is a whole blood analyzer that employs a disposable dry reagent strip containing an electrode and glucose oxidase. Plasma from whole blood diffuses through a porous membrane separating out the erythrocytes. Glucose in the sample is oxidized to glucuronic acid and electrons from this reaction are transferred to the electrode surface through an electrochemical mediator generating a current that is measured by the system. The analytical coefficient of variation (CV) for the PCx is 6.0% at 2.9 mmol/L and 5.0% at 15.9 mmol/L using control solutions. The Vitros 950 method for plasma glucose measurement also uses glucose oxidase but with colorimetric detection. The analytical CV for the Vitros analyzer is 2.7% at 5.3 mmol/L and 1.6% at 15.4 mmol/L using control solutions. The PCx glucose meter is calibrated against the YSI 2300 analyzer whereas the Vitros 950 uses a calibrator for glucose traceable to the National Institute of Standards and Technology (NIST) Standard Reference Material (SRM) 917b. Glucose concentrations are given in SI units and can be converted to conventional units by dividing by 0.055.

### Hematocrit

Hematocrit was measured on the Beckman Coulter HMX hematology analyzer (Beckman Instruments, Brea, CA).

### Statistical analysis

The characteristics of the data were presented using the sample size, median, range and percentages. Comparisons between glucose measurements (RN PCx and PG, LAB PCx and PG, and RN PCx and LAB PCx) were done using the Passing-Bablok regression method [11]. This method makes no assumptions for sample distribution or measurement errors. Pearson correlations were also calculated. A receiver operator characteristic (ROC) curve calculation was performed to establish a cut off value for confirmatory testing. The Passing-Bablok regression, Pearson correlation and ROC curve calculations were done using Microsoft Excel 2000 (Microsoft Corporation, Redmond, WA) with Analyze-it for Microsoft Excel (Analyze-It Software, Ltd., Leeds, England).

Simple and mixed-effect regression analyses were performed in SPSS 13.0 (SPSS Inc., Chicago, IL) to identify variables that affect the agreements of these measurements. Mixed-effect regression analysis was implemented by using the mixed linear model procedure with restricted maximum likelihood (REML) estimation method. This procedure is robust and allows analyses to be performed on data that exhibits correlation and non-constant variability due to repeated measures on the same patients. We assumed an unstructured covariance matrix for all mixed-effect analyses. First, we fitted a model with no covariate to the data (null model). This model was made up of two parts: a fixed part which contains the effect of the overall intercept and a random part, which contains the random effect for the intercept and the within patient residual. Second, we fitted models with covariates to the data including all the predictors as fixed variables and retaining the same random component as in the null model. Model selection and the test for the inclusion of random effects were based on the statistical significance of a chi-squared test for the change in -2 log likelihood between the models with and without the random effects. Third, we estimated the intraclass correlation (ICC), which measures the proportion of the total variance accounted for by between patient variations. We used the approach of Snijder and Bosker [12] to calculate the coefficient of determination (R^2^) as the proportional reduction in the estimated total residual variance from the null model to the model with predictors. For all regression analyses, the dependent variables were the difference between RN PCx and PG, LAB PCx and PG, and RN PCx and LAB PCx. The independent variables included capillary (1 = capillary, 0 = arterial, 0 = venous); arterial (0 = capillary, 1 = arterial, 0 = venous); pipette used to apply blood (1 = yes, 0 = no); sample sent on ice (1 = yes, 0 = no) and group (1 = less than or equal to 4.0 mmol/L, 0 = greater than 4.0 mmol/L). Subgroup regression analyses (≤ 4.0 mmol/L and > 4.0 mmol/L) using hematocrit as the only independent variable was also carried out. A p-value less than .05 was considered to be statistically significant.

## Results

Four hundred and seventy five samples taken from 132 patients were analyzed. The characteristics of the data collected are shown in Table 1. Complete data for all glucose measurements were available on 449 samples. Data missing in other categories ranged from 0.4 to 2.7% except for the time between RN PCx and LAB PCx measurements (5.7%).

**Table 1:** Characteristics of the data collected.

**Characteristics**	**N or median**	**Range or percent**
Patients (n)	132	
Samples collected (n)	475	
Samples per patient (n)	3	1 – 23
Weight (g)	1423	500 – 6609
Corrected Gestational age (weeks)	32	24 – 41
Hematocrit (volume fraction)	0.43	0.188 – 0.692
Glucose meter at the bedside (mmol/L)	4.7	1.4 – 17.5
Glucose meter in the laboratory (mmol/L)	4.2	1.1 – 17.5
Vitros Analyzer (mmol/L)	4.5	1.1 – 17.9
Sample Type (n)		
Capillary	374	(78.7%)
Arterial	82	(17.3%)
Venous	12	(2.5%)
First drop of blood wiped away (n)		
Yes	370	(98.9%)
No	2	(0.5%)
Pipette used to apply blood (n)		
Yes	325	(68.4%)
No	142	(29.9%)
Sample sent on ice (n)		
Yes	464	(97.8%)
No	8	(1.5%)
Time between RN PCx and LAB PCx measurement (min)	26	3 – 110
Glucose meters used at bedside (n)	8	
Glucose meters used in lab (n)	1	
Operators at the Bedside (n)	115	

Median and range values are given for all variable data. Percentages are calculated using the number of samples collected.

**Figure 1:**
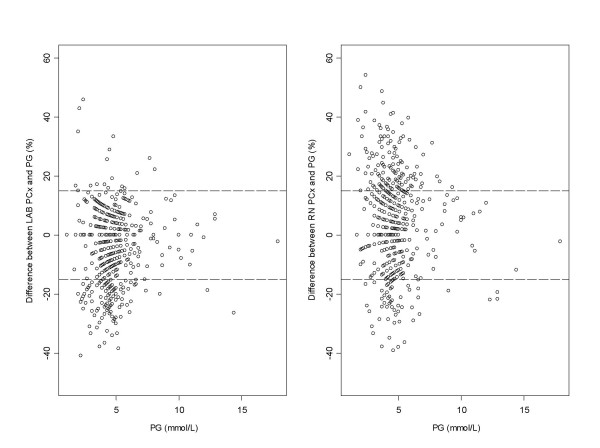
**Comparison between PCx and PG measurements**. Difference plots comparing glucose measurements on the PCx at the bedside (RN PCx) and in the laboratory (LAB PCx) with the corresponding glucose measurements on the laboratory analyser (PG). The two dashed lines represent the total error limits ± 15% .

The Passing-Bablok regression equations and correlations for the blood glucose meter results compared to the laboratory value were: LAB PCx = 1.08 PG – .58, r = .95; RN PCx = 1.18 PG – .55, r = .91. The comparison between the two PCx glucose measurements was RN PCx = .92 LAB PCx – .58, r = .95. Despite these good correlations Figure 1 shows that 42% and 30% of glucose meter results, for RN PCx and LAB PCx compared to PG results respectively, were outside ± 15% total error limits. Furthermore, glucose measurements done on the PCx were significantly different from the PG measurements. The RN PCx results were higher (mean = .20 mmol/L; 95% CI: .06; P < .0001; SD = .81), whereas the LAB PCx results were lower (-.24 mmol/L; 95% CI: .07; P < .001; SD = .63) than the PG reference method. The mean differences were 4.9% and -5.2% for the RN PCx – PG and LAB PCx – PG, respectively.

Further analysis of the data at low concentrations (≤ 4.0 mmol/L, n = 137) showed weaker associations: LAB PCx = 1.19 PG – .74, r = .78; RN PCx = 1.43 PG – 1.11, r = .73. The comparison between the two PCx glucose measurements was RN PCx = 1.16 LAB PCx – .06, r = .83. The proportion of RN PCx and LAB PCx results outside ± 15% error limits were 38.7% and 5.1%, respectively.

### RN PCx – PG

Only two variables showed significance in explaining the difference between the RN PCx values and PG values after adjusting for the effect of other variables (Table 2). Hematocrit had the greatest effect (β = -4.871) on this difference, i.e., for an increase of .01 in hematocrit the difference between RN PCx and PG values decreased by approximately .05 mmol/L. Low glucose concentration also explained some of the difference (β = .2619). On average in the ≤ 4.0 mmol/L group the difference between the RN PCx and the PG value was .27 mmol/L higher than the >4.0 mmol/L group. Overall, the variables in this study were able to explain about 31% of the difference between RN PCx and PG glucose values.

**Table 2:** Mixed-effects regression analyses.

		**RN PCx – PG**			**LAB PCx – PG**			**RN PCx – LAB PCx**		
	**Variables**	**β**	**Std. E**	**P**	**β**	**Std. E**	**P**	**B**	**Std. E**	**P**

**Models with no covariate (null model)**										

**Fixed Effects**	Intercept	.1278	.0553	.023	-.2904	.0418	.000	.4210	.0382	.000
**Random Effects**	Residual	.4286	.0329	.000	.2670	.0200	.000	.5027	.0367	.000
	Intercept	.2190	.0474	.000	.1197	.0255	.000	.0282	.0195	.148
	ICC	.3382			.3095			.0531		

**Models with the effects of Covariates**										

**Fixed Effects**	Intercept	1.7615	.5606	.002	1.552	.3135	.000	.3695	.5794	.524
	Arterial sample	.3230	.2303	.161	-.0450	.1584	.776	.3270	.2519	.195
	Capillary sample	.1648	.2209	.456	-.6092	.1469	.000	.8918	.2441	.000
	Pipette used to apply blood	-.0993	.0918	.280	-	-	-	-.2478	.1003	.014
	Sample sent on ice	.3414	.2947	.247	-	-	-	.2270	.3248	.485
	Time between bedside and laboratory glucose measurement	.0022	.0015	.134	.0013	.0010	.204	.0016	.0016	.330
	Weight (divided by 100)	.0047	.0061	.443	-.0039	.0040	.330	.0072	.0061	.239
	Corrected gestational age	-.0049	.0154	.751	.0065	.0100	.519	-0.122	.0156	.435
	Hematocrit	-4.871	.4283	.000	-3.5710	.2754	.000	-1.3164	.4338	.003
	Group (≤ 4 mmol/L)	.2619	.0744	.000	.2392	.0505	.000	.0271	.8034	.735
**Random Effects**	Residual	.3956	.0320	.000	.1974	.0151	.000	.5053	.0393	.000
	Intercept	.0532	.0239	.025	.0111	.0080	.167	.0150	.0189	.429
	ICC	.1185			.0532			.0288		
	R-Squared	.3070			.4608			.0200		

The analyses included fixed-effects and random-effects parameter estimates (β); approximate standard errors (Std. E) and p-values (P) for the difference between PCx and PG measurements taking into account all variables collected. RN, registered nurse (bedside glucose meter); LAB (laboratory glucose meter); and PG, plasma glucose (Vitros 950).

### LAB PCx – PG

The three significant variables that explained part of the variation in the difference between the LAB PCx values and the PG values after adjusting for the effect of other variables were the capillary sample, group (≤ 4.0 mmol/L) and hematocrit (Table 2). Hematocrit had the greatest effect (β = -3.5710) on this difference, i.e., for an increase of .01 in hematocrit the difference between LAB PCx and PG values decreased by approximately .04 mmol/L. In the ≤ 4.0 mmol/L group the difference between the LAB PCx and the PG value was on average .24 mmol/L (β = .2392) higher compared to the > 4.0 mmol/L group. The average difference between LAB PCx and PG values was .61 mmol/L lower (β = .6092) if a capillary sample was used compared to an arterial or venous sample. Overall, the variables in this study were able to explain about 46% of the difference between LAB PCx and PG glucose values.

### RN PCx – LAB PCx

The difference between the PCx values obtained at the bedside and the PCx values obtained in the laboratory, after adjusting for the effect of other variables, can be explained in part by three variables, capillary sample (β = .8918), pipette used to apply the sample (β = -.2478), and hematocrit (β = -1.316)(Table 2). The average difference between RN PCx and LAB PCx values was .89 mmol/L higher than if a capillary sample was used compared to an arterial or venous sample. If a pipette was used to apply sample to the test strip at the bedside the difference between the two measurements was on average .25 mmol/L lower than if no pipette was used. Hematocrit had the greatest effect with an average difference between RN PCx and LAB PCx values of approximately .01 mmol/L per .01 unit increase in hematocrit. Overall, these variables and the other variables used in this study, explained only 3% of the difference between RN PCx and LAB PCx glucose values.

### Effect of repeated measures on patients and difference in operators

Since samples were often collected more than once per patient (1 to 23) the effect of repeated measures on the total variation was determined. The ICC values showed the variation within patients accounted for only a minor portion of the total variation in the differences between measures (Table 2). The highest ICC was seen for RN PCx – PG (ICC = .1185) with lower ICC values for LAB PCx – PG (ICC = .0532) and RN PCx – LAB PCx (ICC = .0288).

There were 115 different nurses who performed the bedside glucose measurement. The ICC values were small for all comparisons indicating minimal nurse specific effects on the analysis [see Additional File 1].

### Effect of hematocrit

**Figure 2:**
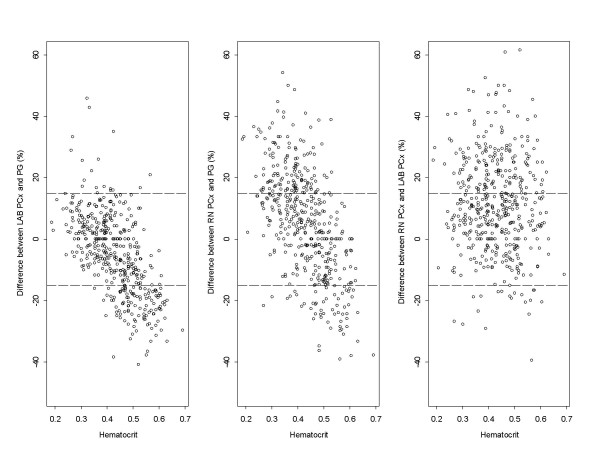
**Effect of hematocrit on PCx measurements**. Effect of hematocrit on the difference in glucose measurements on the PCx glucose meter performed at the bedside (RN PCx) and in the laboratory (LAB PCx) compared to the laboratory analyzer (PG).

Of all the variables in this study, hematocrit had the greatest effect on the differences in glucose values between the three measurements made. Figure 2 shows the negative correlation between hematocrit and the difference between the PCx and PG measurements. The data points cross over the zero difference showing that glucose differences are positive if the hematocrit is lower and negative when the hematocrit is higher. An additional mixed-effect regression analysis was performed to see whether the level of glucose concentration had an impact on the extent to which hematocrit contributed to the difference between measurements. This analysis showed that the R-square was consistently higher in the ≤ 4 mmol/L group compared to the > 4 mmol/L group (Table 3). Thus, hematocrit was a greater contributor to the difference between measurements at lower glucose concentrations.

**Table 3:** Mixed effect regression with only hematocrit as the independent variable.

		**RN PCx – PG**			**LAB PCx – PG**			**RN PCx – LAB PCx**		
	Variables	ALL	PG ≤ 4 mmol/L	PG > 4 mmol/L	ALL	PG ≤ 4 mmol/L	PG > 4 mmol/L	ALL	PG ≤ 4 mmol/L	PG > 4 mmol/L

**Model with no covariate (null model)**										

**Fixed Effects**	Intercept	.1278 (.023)	.2664 (.000)	.0743 (.292)	-.2904 (.000)	-.1427 (.003)	-.3441 (.000)	.4210 (.000)	.4055 (.000)	.4334 (.000)
**Random Effects**	Residual	.4286 (.000)	.1491 (.000)	.5294 (.000)	.2670 (.000)	.1057 (.000)	.3178 (.000)	.5027 (.000)	.1760 (.000)	.6344 (.000)
	Intercept	.2190 (.000)	.1748 (.000)	.2638 (.000)	.1197 (.000)	.0910 (.000)	.1498 (.000)	.0282 (.148)	.0563 (.061)	.0274 (.349)
	ICC	.3382	.5397	.3326	.3095	.4626	.3204	.0531	.2424	.0414

**Models with the effects of hematocrit**										

**Fixed Effects**	Intercept	2.1556 (.000)	2.1616 (.000)	2.2962 (.000)	1.3237 (.000)	1.1849 (.000)	1.5383 (.000)	.9019 (.000)	1.0007 (.000)	.8481 (.000)
	Hematocrit	-4.5545 (.000)	-4.1764 (.000)	-5.0828 (.000)	-3.6237 (.000)	-2.9218 (.000)	-4.3113 (.000)	-1.1054 (.005)	-1.3189 (.006)	-.9837 (.068)
**Random Effects**	Residual	.4061 (.000)	.1390 (.000)	.5052 (.000)	.2588 (.000)	.1040 (.000)	.3039 (.000)	.4970 (.000)	.1714 (.000)	.6306 (.000)
	Intercept	.05437 (.013)	.0287 (.204)	.0621 (.063)	.01226 (.176)	.0145 (.262)	.0089 (.461)	.0265 (.172)	.0499 (.081)	.02625 (.383)
	ICC	.1181	.1711	.1095	.0452	.1224	.0285	.0506	.02255	.0400
	R-Squared	.2890	.4822	.2848	.2990	.3976	.3311	.0139	.0474	.0075

The analyses included fixed-effects and random-effects parameter estimates (p-values) for the difference between PCx and PG measurements for according to all, low (≤ 4 mmol/L) and high (> 4 mmol/L) glucose concentration. RN, registered nurse (bedside glucose meter); LAB (laboratory glucose meter); and PG, plasma glucose (Vitros 950).

### Hypoglycemia cut off value

**Figure 3:**
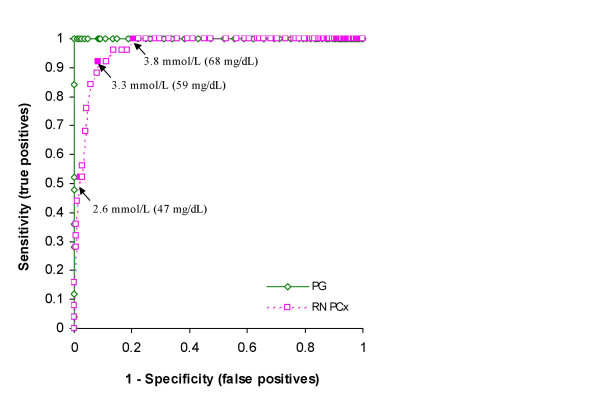
**ROC curve for neonatal hypoglycemia**. ROC curve for plasma glucose measured on the Vitros 950 (PG) and whole blood glucose measured at the bedside with the PCx glucose meter (RN PCx). The arrows indicate the PCx screening cut off values for hypoglycemia of 2.6 mmol/L (suggested hypoglycemic screening threshold value for PG), 3.3 mmol/l (highest accuracy) and 3.8 mmol/L (highest sensitivity). The diagonal line indicates no discrimination.

There were 25 cases where the PG value was < 2.6 mmol/L. However, only 13 of these cases had a bedside PCx result < 2.6 mmol/L. The ROC curve in Figure 3 shows a sensitivity of only 52.0% and a specificity of 97.9% at this cut off for a positive predictive value (PPV) of 59.1%. The cut off with the highest accuracy (sensitivity = 92%, specificity = 91.7%) was 3.3 mmol/L. As the cut off value increases the sensitivity of the PCx for detecting hypoglycaemia increases. All hypoglycemic cases (PG < 2.6 mmol/L) would be detected using an RN PCx cut off of 3.8 mmol/L at the expense of increasing the number of false positive results from 9 to 88 (PPV = 22.1%). In practical terms, for approximately every five samples with an RN PCx value < 3.8 mmol/L sent for confirmatory testing one sample would have a PG value < 2.6 mmol/L indicating hypoglycemia.

## Discussion

In this study glucose concentrations were found to be higher when measured at the bedside (RN PCx) compared to when the measurements were performed in the laboratory (LAB PCx or PG). The difference between glucose meter results, either at the bedside or in the laboratory, compared to the laboratory analyzer ranged from -29% to 39% and -32% to 21%, respectively. Only 58% of the all RN PCx measurements and 39% at low values (≤ 4 mmol/L) achieved a total error difference of 15% of the PG values. A 15% error limit is quite a liberal quality specification that should be achievable and has been suggested previously by the ADA and other groups [10]. A 15% error on a value of 2.6 mmol/L is .4 mmol/L or a range between 2.2 to 3.0 mmol/L, which will significantly affect clinical management. If the desired 5% error limit is applied the range is reduced from 2.5 to 2.7 mmol/L. A rational error estimate however depends on what can be achieved analytically (method variation) and biologically (variation around a homeostatic set point). The concept of allowable total error (TEa) combines analytical and biological variation. A frequently quoted TEa for glucose is 6.9% [13]. Therefore, for a hypoglycemic threshold value of 2.6 mmol/L the practical error around this value is closer to 2.4 to 2.8 mmol/L. The definition of neonatal hypoglycemia must take into consideration the broad error limits of glucose measurements, particularly when less accurate glucose meters are used.

The poor accuracy of the PCx resulted in low sensitivity for detecting hypoglycemia at the bedside. Approximately half of the cases with laboratory glucose values less than the operational threshold for hypoglycemia of < 2.6 mmol/L were missed using this threshold for the glucose meter. Recognizing that the highest sensitivity for detecting hypoglycemia is necessary to avoid neurological sequelae in both asymptomatic and symptomatic neonates, the pertinent question is at what screening cut off value should a blood sample be drawn and sent for confirmatory testing? Increasing the screening threshold value on the glucose meter increases the number of true positive cases at the expense of including more false positive cases. To achieve 100% sensitivity for detecting hypoglycemia, one in four of all samples screened in this study would require confirmatory testing based on a screening cut off value of 3.8 mmol/L. The poor accuracy of glucose meters for detecting neonatal hypoglycemia is well known and varies between glucose meters and study populations [9,14]. Although this limitation attenuates the advantage of bedside glucose monitoring with respect to time, this method still reduces the number of larger blood volume collections required for glucose testing in the laboratory. The cut off value chosen for confirmatory screening will be dependent on the glucose meter and the comparator reference method. If NICUs have access to POC blood gas analyzers with glucose modules (accuracy is comparable to laboratory methods), the time delay for confirmatory analysis would be reduced compared to sending the sample to the laboratory for analysis.

Many variables have been shown to contribute to the inaccuracy of glucose meters, but no study has incorporated as many variables in a large mixed-effects analysis as this present study. We found that only 31% of the variance between the glucose meter at the bedside and laboratory analyzer results could be attributed to the measured variables. Some of the unexplained variance may be due to differences in calibration between the PCx glucose meter and the Vitros analyzer. Surprisingly, and in contrast to other studies, we found that neither time between bedside and laboratory analysis, nor transport on ice were significant variables in explaining the observed difference. The fall in glucose concentration between bedside and laboratory analysis suggests glycolysis had occurred, but this was not supported by the time variable (not significant). The rate of cord blood glycolysis 30 minutes post draw at room temperature has been found to be about .33 mmol/L/h [15]. This is quite substantial, but in our study blood was kept on ice and analyzed in less than one hour (mean = 35.2 min, CI = 2.1 min). Although the activity of the neonatal red blood cell glycolytic enzymes such as pyruvate kinase are higher in neonate than in adult red blood cells [16], enzyme kinetics are most likely to change from a rapid to a reduced metabolizing state because of the sudden drop in temperature and termination of glucose supply. No detailed time studies have been done to examine the kinetics of glycolysis immediately following blood withdrawal and cooling on ice. Rapid glycolysis suggested by higher bedside glucose value could have implications for the definition of an operational threshold and the management of neonatal hypoglycemia.

Hematocrit was the largest and most consistent variable accounting for the observed difference in glucose results in all models compared. The manufacturer claims there is no significant effect with hematocrits between .2 and .7. However, it is not clear from the product information material if this range is also applicable for neonatal samples. All samples in this study had hematocrits essentially within this range (.19 to .69). The hematocrit effect using filter-based test strips is well known (including the PCx glucose meter), although not all glucose meters have this problem [10]. Separation of plasma from cells for glucose measurement occurs by membrane filtration in contrast to the conventional method of centrifugation. The rate of filtration and the volume filtered depends on the properties of the sample. If the volume of plasma filtered varies then the glucose concentration will vary since it is a function of the number of molecules per volume. A sample with more red blood cells will yield less plasma water then a sample with fewer red blood cells because of the fixed analysis time between sample application and result readout. The hematocrit effect was greater in samples containing lower (≤ 4.0 mmol/L) than in higher (> 4.0 mmol/L) glucose concentrations since proportionally a change in volume will have a more pronounced effect at low concentrations.

In essence, any variable that affects the flow of plasma through the filter membrane will alter the glucose concentration. These rheological factors may include the size and shape of neonatal red blood cells, microclot formation in samples, contamination by interstitial fluid, hemolysis, altered protein quantity [17], protein deposition, fibrin aggregation, platelet or other cellular phenomena triggered by the test strip [10]. Interestingly, capillary samples were identified as a factor in the difference between LAB PCx and PG values but not between RN PCx and PG values. This may be due to greater hemolysis occurring post RN PCx analysis for a capillary specimen compared to a venous or arterial sample. Accelerated aggregation of red blood cells may result in more plasma water passing through the filter membrane. Aggregation of red blood cells can be triggered by free radicals and antioxidants through activated polymorphoneutrophils [18]. One study demonstrated increased oxidants to antioxidant ratios in infants within the first 72 hours of birth [19]. The neonatal red blood cell is also structurally different from adult red blood cells [20]. Neonatal red blood cells have a larger cell volume and hemoglobin concentration than do adult red blood cells. They are also more fragile [21] and thus may be broken more readily when applied to the barbed-like mesh of a filter membrane. Furthermore, drugs and altered pH can affect the measurement of glucose using strip-based glucose meters [10]. One or more of these unmeasured variables may have contributed to the observed difference between the glucose meter values and the laboratory values.

## Conclusion

Our results show that screening for neonatal hypoglycemia using the PCx glucose meter provides an estimate of blood glucose but it also requires confirmation with the more accurate and precise laboratory analyzer. The optimal screening cut off for confirmatory testing is dependent on what is an acceptable PPV. Hematocrit and low glucose concentration are the most significant variables contributing to the difference between bedside glucose meter and laboratory analyzer measurements.

## Competing interests

The author(s) declare that they have no competing interests.

## Authors' contributions

VG and SB conceived the study, and participated in its design and coordination. WS coordinated participant recruitment, sample collection and questionnaire design. CB participated in the study design and data analysis. AI and CB performed the statistical analysis. CB, VG and AI prepared the manuscript. All authors read and approved the final manuscript.

## Pre-publication history

The pre-publication history for this paper can be accessed here:



## Supplementary Material

Additional File 1 **Mixed-effects regression analysis results: effect of nurses on comparison of glucose measurements**. The analyses included fixed-effects and random-effects parameter estimates (β) and p-values (P) for the difference between PCx and PG measurements taking into account all variables collected. RN, registered nurse (bedside glucose meter); LAB (laboratory glucose meter); and PG, plasma glucose (Vitros 950). Click here for file

## References

[B1] Deshpande S, Platt MW (2005). The investigation and management of neonatal hypoglycaemia. Semin Fetal Neonatal Med.

[B2] Cornblath M, Schwartz R, Aynsley-Green A, Lloyd JK (1990). Hypoglycemia in infancy: the need for a rational definition. A Ciba Foundation discussion meeting. Pediatrics.

[B3] Cornblath M, Hawdon JM, Williams AF, Aynsley-Green A, Ward-Platt MP, Schwartz R, Kalhan SC (2000). Controversies regarding definition of neonatal hypoglycemia: suggested operational thresholds. Pediatrics.

[B4] Stanley CA, Baker L (1999). The causes of neonatal hypoglycemia. NEJM.

[B5] Canadian Paediatric Society (2004). Screening guidelines for newborns at risk for low blood glucose. Paediatr Child Health.

[B6] American Diabetes Association (1996). Self-monitoring of blood glucose consensus statement. Diabetes Care.

[B7] International Organization for Standardization (2003). Requirements for in vitro blood glucose monitoring systems for self-testing in managing diabetes mellitus.

[B8] Kost GJ, Vu HT, Lee JH, Bourgeois P, Kiechle FL, Martin C, Miller SS, Okorodudu AO, Podczasy JJ, Webster R, Witlow KJ (1998). Multicentre study of oxygen-insensitive handheld glucose point-of-care testing in critical care/hospital/ambulatory patients in the United States and Canada. Crit Care Med.

[B9] Ho HT, Yeung WKY, Young BWY (2003). Evaluation of "point of care" devices in the measurement of low blood glucose in neonatal practice. Arch Dis Child Fetal Neonatal Ed.

[B10] St-Louis P, Ethier J (2002). An evaluation of three glucose meters and their performance in relation to criteria of acceptability for neonatal specimens. Clin Chim Acta.

[B11] Passing H, Bablok W (1983). A new biometrical procedure for testing the equality of measurements from two different analytical methods. J Clin Chem Clin Biochem.

[B12] Snijders TAB, Bosker RJ (1999). Multilevel analysis: An introduction to basic and advanced multilevel modeling.

[B13] Desirable Specifications for total error, imprecision and bias, derived from biologic variation. http://www.westgard.com/biodatabase1.htm.

[B14] Girouard J, Forest J-C, Masse J, Leroux M, Bradburn NC, Noblet TC, Joynes JO, Baum J (2000). Multicenter evaluation of the glucometer elite XL meter, an instrument specifically designed for neonates. Diabetes Care.

[B15] Lin YL, Smith CH, Dietzler DN (1976). Stabilization of blood glucose by cooling with ice: an effective procedure for preservation of samples from adults and newborns. Clin Chem.

[B16] Mohrenweiser HW, Fielek S, Wurzinger KH (1981). Characteristics of enzymes of erythrocytes from newborns and adults: activity, thermostability, and electrophoretic profile as a function of cell age. Am J Hematol.

[B17] Rampling MW, Wittingstall P, Martin G, Bignall S, Rivers RP, Lissauer TJ, Bailey PC (1989). A comparison of the rheologic properties of neonatal and adult blood. Pediatr Res.

[B18] Baskurt O, Meiselman HJ (1998). Activated polymorphonuclear leukocytes affect red blood cell aggregability. J Leukoc Biol.

[B19] Ochoa JJ, Ramirez-Tortosa MC, Quiles JL, Palomino N, Robles R, Mataix J, Huertas JR (2003). Oxidative stress in erythrocytes from premature and full-term infants during their first 72 h of life. Free Radic Res.

[B20] Matovcik LM, Mentzer WC, Schrier SL (1985). The membrane of the human neonatal red cell. The red blood cell membrane.

[B21] Meyburg J, Bohler T, Linderkamp O (2000). Decreased mechanical stability of neonatal red cell membrane quantified by measurement of the elastic area compressibility modulus. Clin Hemorheol Microcirc.

