# Supplementary material for: Screening for hypoglycemia at the bedside in the neonatal intensive care unit (NICU) with the Abbott PCx glucose meter
Source: BMC Pediatr. 2006 Nov 3;6:28. doi: 10.1186/1471-2431-6-28 (PMC1660538; doi:10.1186/1471-2431-6-28)
Supplement: Additional File 1 — Mixed-effects regression analysis results: effect of nurses on comparison of glucose measurements. The analyses included fixed-effects and random-effects parameter estimates (β) and p-values (P) for the difference between PCx and PG measurements taking into account all variables collected. RN, registered nurse (bedside glucose meter); LAB (laboratory glucose meter); and PG, plasma glucose (Vitros 950). [file 1471-2431-6-28-S1.doc]

| Variables | **RN PCx - PG** | | **LAB PCx - PG** | | **RN PCx – LAB PCx** | |
| --- | --- | --- | --- | --- | --- | --- |
|  | **β** | **P** | **β** | **P** | **β** | **P** |
|  |  |  |  |  |  |  |
| **Intercept** | 1.9621004 | .001 | 1.5386943 | .000 | .5177712 | .385 |
| **Pipette used to apply blood** | -.1250991 | .229 | - | - | -.2612178 | .015 |
| **Capillary sample** | .0261663 | .926 | -.5936803 | .000 | .5179251 | .090 |
| **Arterial sample** | .2663776 | .280 | -.0366903 | .818 | .2673187 | .312 |
| **First drop of blood wiped away** | .1754057 | .379 | - | - | .4294828 | .043 |
| Sample sent on ice | .3668645 | .280 | - | - | .1959457 | .589 |
| **Group (≤4.0 mmol/L)** | .2685793 | .000 | .2467084 | .000 | .0251072 | .754 |
| **Time between bedside and laboratory glucose measurement** | .0024288 | .118 | .0014043 | .165 | .0010762 | .518 |
| **Weight** | .0000533 | .345 | -.0000412 | .268 | .0000972 | .106 |
| **Corrected gestational age** | -.0110104 | .438 | .0071309 | 454 | -.0164889 | .275 |
| **Hematocrit** | 4.9821210 | .000 | 3.6554990 | .000 | 1.3898722 | .001 |
| **Residual** | .4112877 | .000 | .1982218 | .000 | .4862908 | .000 |
| **Between nurses variance** | .0377662 | .050 | .0104634 | .241 | .0219128 | .177 |
| **ICC** | 0.084 | - | 0.050 | - | 0.043 |  |
